# Functional Study of miR-27a in Human Hepatic Stellate Cells by Proteomic Analysis: Comprehensive View and a Role in Myogenic Tans-Differentiation

**DOI:** 10.1371/journal.pone.0108351

**Published:** 2014-09-29

**Authors:** Yuhua Ji, Jinsheng Zhang, Wenwen Wang, Juling Ji

**Affiliations:** 1 Key Laboratory of Neuroregeneration, Nantong University, Nanton, China; 2 Department of Pathology, Shanghai Medical College, Fudan University, Shanghai, PR China; 3 Department of Pathology, Medical School of Nantong University, Nantong, PR China; Georgia Regents University, United States of America

## Abstract

We previous reported that miR-27a regulates lipid metabolism and cell proliferation during hepatic stellate cells (HSCs) activation. To further explore the biological function and underlying mechanisms of miR-27a in HSCs, global protein expression affected by overexpression of miR-27a in HSCs was analyzed by a cleavable isotope-coded affinity tags (cICAT) based comparative proteomic approach. In the present study, 1267 non-redundant proteins were identified with unique accession numbers (score ≥1.3, i.e. confidence ≥95%), among which 1171 were quantified and 149 proteins (12.72%) were differentially expressed with a differential expression ratio of 1.5. We found that up-regulated proteins by miR-27a mainly participate in cell proliferation and myogenesis, while down-regulated proteins were the key enzymes involved in de novo lipid synthesis. The expression of a group of six miR-27a regulated proteins was validated and the function of one miR-27a regulated protein was further validated. The results not only delineated the underlying mechanism of miR-27a in modulating fat metabolism and cell proliferation, but also revealed a novel role of miR-27a in promoting myogenic tans-differentiation during HSCs activation. This study also exemplified proteomics strategy as a powerful tool for the functional study of miRNA.

## Introduction

microRNAs (miRNAs) regulate gene expression post-transcriptionally by binding primarily to the 3′untranslated region (3′UTR) of their target mRNAs, resulting in mRNA destabilization or translational repression[1]. Genes encoding 2042 mature human miRNAs have so far been identified (miRBase v.19) [2] and miRNAs are predicted to regulate the expression of up to 60% of human protein-encoding genes [3]. The best way to understand the biological function of a miRNA is to identify the genes that it regulates. Several bioinformatics methods have been developed for miRNA target prediction, including TargetScan (www.targetscan.org), miRanda (www.microrna.org), TarBase (diana.cslab.ece.ntua.gr), PicTar (pictar.mdcberlin. de) et al. However since the mechanism of miRNA target recognition is still not fully understood, target gene prediction is not accurate and sometimes over predict [4]. In addition, a single miRNA can target hundreds of proteins and a single protein can be influenced by multiple miRNAs [5]. Thus comprehensive understanding of the phenotypic effects of miRNAs at the cellular level is currently difficult.

The use of quantitative proteomic strategies to characterize targets of miRNAs has opened new avenues to miRNA biology study [6]. The method of cleavable isotope-coded affinity tags (cICAT) coupling with nano LC-MS/MS is a quantitative proteomic approach that enables rapid, comprehensive and reliable analysis of the proteomes of two comparable samples [7]. More importantly, compared with other quantitative proteomic strategies, cICAT based approach could greatly reduce the sample complexity, therefore those low abundance proteins could be readily identified.

We have previously reported that miR-27a,b suppresses fat accumulation and promotes cell proliferation during hepatic stellate cells (HSCs) activation [8]. Thereafter, miR-27 has been evidenced to act as negative regulator of adipocyte differentiation [9] or lipid metabolism [10], and positive regulator of cell proliferation [11] by several groups. It has also been regarded as an oncogene in some malignant tumor [12], [13]. To further explore the possible functions and underlying mechanism of miR-27a during HSCs activation, human stellate cell line LX2/miR-27a stable transfectants was established and validated. Global protein expression profiles were compared between LX2/miR-27a and LX2/miR-neg control by cICAT-based proteomic approach. We found that out of 1267 identified proteins, 149 proteins were differentially expressed, and 75 were repressed by miR-27a overexpression among which, 15 proteins were predicted miR-27a targets. The bio-significance of miR-27a was analyzed based on the functional annotation of miR-27a regulated proteins. Individual siRNA mediated knock-down of one miR-27a regulated protein was performed to demonstrate the phenotypic effects.

## Materials and Methods

### 1. Plasmid constructions

To construct miRNA expression plasmid, miR-27a expression fragments designed according to manufactures’ instructions, miR-27a, sense 5′-TGCTGTTCACAGTGGCTAAGTTCCGCGTTTTGGCCACTGACTGACGCGGAACTGCCACTGTGAA-3′, anti-sense 5′-CCTGTTCACAGTGGCAGTTCCGCGTCAGTCAGTGGCCAAAACGCGGAACTTAGCCACTGTGAAC-3′; were cloned into pcDNA6.2-GW/EmGFP-mir vector (Invitrogen, Carlsbad, CA) after annealing the oligonucleotides, termed as pcDNA6.2-GW/EmGFP-mir-27a. The pcDNA6.2-GW/EmGFP-mir-neg vector was provided by Invitrogen. DNA sequencing analyses confirmed the nucleotide sequences of the constructed plasmids.

### 2. Establishment of stable transfectants

Human hepatic stellate cell line LX2 cells [14] were maintained in DMEM (Invitrogen), supplemented with 10% FBS (Invitrogen), and were incubated in a humidified atmosphere of 5% CO_2_ and 95% air at 37°C. The medium was changed every 48 hours. Stable transfectants were constructed using LX2 cells that had been plated at approximately 1×10^5^ per 60-mm diameter culture dish and cultured overnight. The cells were transfected with 5 µg pcDNA6.2-GW/EmGFP-mir-27a or mir-neg control plasmids by Lipofectamine 2000 (Invitrogen). Transfection efficiencies were checked by EmGFP expression under fluorescent microscope. Clones were selected and maintained in DMEM supplemented with 10 µg/ml Blasticidin (Invitrogen). Two stably transfected cell lines, LX2/miR-27a and LX2/miR- neg were isolated after 28 days’ selection.

### 3. Real-time reverse transcription PCR (RT-PCR)

Total RNA from LX2 cells was extracted using Trizol reagent (Invitrogen). cDNAs and the first-strand cDNAs of miRNA were produced according to the manufacturer’s instructions for Thermoscript RT-PCR system (Invitrogen) or NCode miRNA First-Strand cDNA Synthesis kits (Invitrogen). For the quantitative detection of miR-27a and mRNAs of interested genes, the templates and primer sets (Table S1) were mixed with SYBR qPCR master mix (TaKaRa, Dalian, China), and real-time PCR was performed using Rotor-Gene 3000 (Corbett Research, Sydney). The cycling parameters were: initial denaturing at 94°C for 15 sec, followed by 40 cycles of 94°C denaturing for 10 sec, primer annealing and extension at 60°C for 40 sec. To ensure the specificity of the amplification reaction, melting curve analysis was performed. The expression of miR-27a was normalized to U6snRNA, and mRNAs were normalized to GAPDH. Relative gene expression was presented by comparative CT method.

### 4. Quantitative proteomic analysis

Global protein expression profile changes of LX2/miR-27a transfectants were analyzed by a cleavable isotope-coded affinity tags (cICAT) labeling coupled with online 2D nanoLC-MS/MS based quantitative proteomic approach. cICAT reagents were from Applied Biosystems (Foster City, CA).

#### (A) cICAT labeling

Proteins from LX2/miR-27a and LX2/miR-neg control were labeled with isotopically heavy (H) and light (L) cICAT reagents respectively following the manufacture’s protocol. Briefly 100 µg total protein collected from LX2/miR-27a and negative control LX2/miR-neg were labeled, respectively, with isotopically light (^12^C for LX2/miR-neg) and heavy (^13^C for LX2/miR-27a ) cICAT reagents at 37°C for 2 hours. The labeled preparations were combined and digested with trypsin (Promega, madison, WI) overnight at 37°C using an enzyme-to-protein ratio of 1∶50 w/w. The resulting peptides were subsequently purified by cation exchange chromatography and avidin affinity chromatography (Applied Biosystems). The biotin group on the tag was removed by acid cleavage and the peptides were dried by vacuum-evaporation using a Speedvac™ system (Thermo Scientific).

#### (B) 2D nanoLC-MS/MS analysis

The dried peptides were resuspended in 80 ul of an aqueous solution containing 0.1% FA and 5% acetonitrile, the resulting solution was loaded onto a 30*0.5 mm strong cation exchange column (Agilent Technologies) and separated into 17 fractions with a step gradient of 0 mM, 10 mM, 20 mM, 30 mM, 40 mM, 50 mM, 60 mM, 70 mM, 80 mM, 90 mM, 100 mM, 125 mM 150 mM, 200 mM, 300 mM, 400 mM, 500 mM and 900 mM, 0.1% FA, 5% acetonitrile. The elutions from SCX column were further separated on a 150*0.075 mm Vydac C18 reverse phase column (Grace, inc) in line after a nanotrap column (Agilent Technologies) using a nanoHPLC 1100 system (Agilent Technologies). Separation of the peptides was performed at 400 nl/min and was coupled to online analysis by tandem mass spectrometry using a QstarXL MS/MS system (Applied Biosystems) equipped with a nanospray ion source (Applied Biosystems). Elution of the peptides into the mass spectrometer was performed with a linear gradient from 95% mobile phase A (0.1% FA, 99.9% water) to 35% mobile phase B (0.1% FA, 99.9% acetonitrile) over 120 minutes followed by 80% mobile phase B for 10 min. The peptides were detected in positive ion mode using an IDA (information dependent acquisition) method in which three most abundant ions detected in a MS scan were selected for MS/MS analysis. Two independent analyses were performed.

#### (C) Data Analysis

For protein identification and quantification, all MS/MS spectra were searched against the IPI human protein database (V3.83) using ProteinpilotTM 3.0.1 (Applied Biosystem). The software compares relative intensity of proteins present in samples based on the intensity of reporter ions released from each labeled peptide and automatically calculates protein ratios and p-values for each protein. For protein identification, 95% confidence was used and the corresponding FDR <1%.

### 5. Bio-functional analysis of differentially expressed proteins

GOfact (http://61.50.138.118/gofact/cgi/gofact2009.cgi) strategy [15], [16] which based on the structured and controlled vocabularies - Gene Ontology (GO), and the GO annotation from related databases was used to identify the functional distribution and the enriched functional categories of miR-27a regulated proteins in LX2 cells. The subcellular locations and bio-functions of proteins were also annotated by Protein Knowledgebase (UniprotKB) (http://www.uniprot.org/).

### 6. Transfection of siRNA

Transfection of siRNA was performed according to the manufacturer’s protocol (Sigma, Saint Louis, MO). LX2 and LX2/miR-27a transfectants cultured in 24-well plates or 6-cm dishes were transfected at 50–70% confluence with siRNA targeting human four and a half LIM domains 1 (FHL1) by means of the siRNA transfection reagent RNAiMAX (Invitrogen). NTC (Non-targeting control) siRNA was transfected simultaneously as negative control. After 48 h transfection, the efficiency of siRNA-mediated mRNA degradation was assessed by real-time RT–PCR.

### 7. Proliferation and migration assays

The effects of siRNA transfection on LX2/miR-27a transfectants migration were measured by using a modified Boyden chamber assay. Two days after transfection, 2×10^4^ cells in serum-free DMEM were plated on the upper chamber of each Transwell (Costar, Corning Inc., NY) with 8 µm pores, while the lower chamber contained 800 µl completed medium. Transfected cells were incubated for 16 h at 37°C in 5% CO2. Non-migrating cells were carefully removed from the upper surface of the membrane with cotton swabs. Membranes were stained with crystal violet and mounted onto glass slides. Migration was quantified by counting cells in eight 200x microscopic fields.

Forty-eight hours after siRNAs transfection, the cell proliferation of LX2 cells was detected by incorporation of 5-ethynyl-2′-deoxyuridine (EdU) with the Cell-Light EdU Apollo 567 Cell Proliferation Kit (Ruibo Biotech, Guangzhou, China). According to the kit’s protocol, cells were incubated with 10 µM EdU for 16 h before fixation, permeabilization, and EdU staining. EdU was detected by Apollo fluorescent dye at 567 nm wave length and nuclei were counterstained with 5 µg/ml Hoechst 33342. For each well, eight fields were counted at a 200x magnification. The results were expressed as the labeling index according to the following formula: number of EdU-positive nuclei×100/number of total nuclei.

### 8. Statistics assay

Data are expressed as the mean ± SD. Comparison between groups were made by Student’s t test (two tailed) or one-way ANOVA followed by Tukey's multiple comparison test. The relationship between two data sets was analyzed by linear regression. Differences were considered significant if P<0.05. Unless otherwise specified, all assays were performed in triplicate.

## Results and Discussion

### 1. Biological characterization of LX2/miR-27a stable transfectants

**Figure 1:**
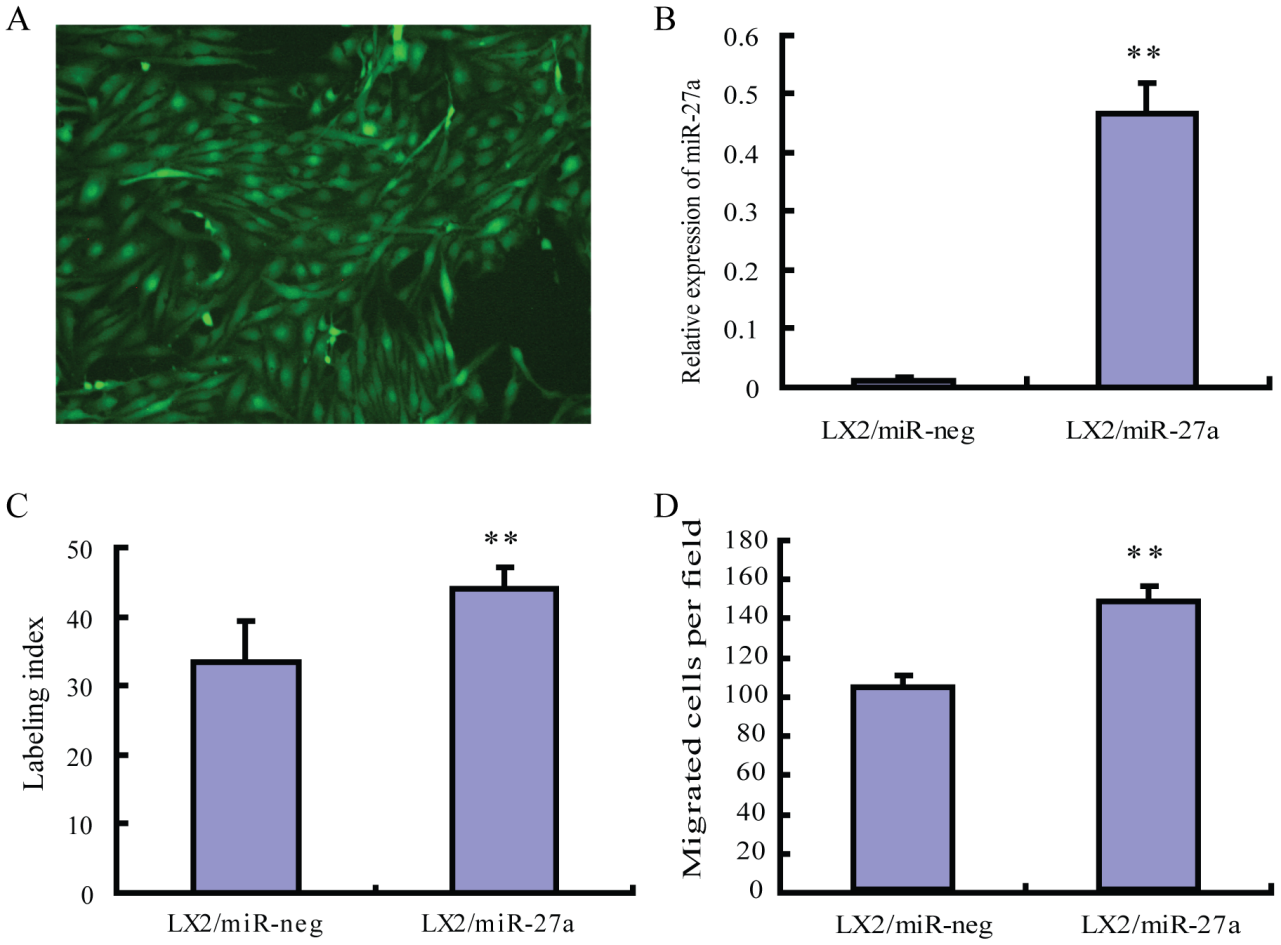
Establishment and biological characters of LX2/miR-27a, LX2/miR-neg stable transfectants. (A) Almost all cells in the positive clone expressed EmGFP (green), original magnification ×200. (B) The expression of miR-27a in LX2/miR-27a, LX2/miR-neg stable transfectants. (C) Over-expression of miR-27a promoted LX2 cell proliferation. (D) miR-27a over-expression facilitated LX2 migration. **P<0.01 compared with LX2/miR-neg.

To explore the biological effects of miR-27a overexpression on HSCs, we established a LX2/miR-27a stable transfectants (Figure 1A). The expression of mature miR-27a increased significantly in LX2/miR-27a stable transfectants (Figure 1B). As it was expected, LX2/miR-27a stable transfectants showed increased cell proliferation and migration compared to LX2/miR- neg stable transfectants (Figure 1C and D). The influence of miR-27a over expression on lipid metabolism was not measurable due to the already activated HSC phenotype of LX2 cell line.

### 2. Identification of miR-27a regulated proteins by cICAT-based proteomic analyses

**Figure 2:**
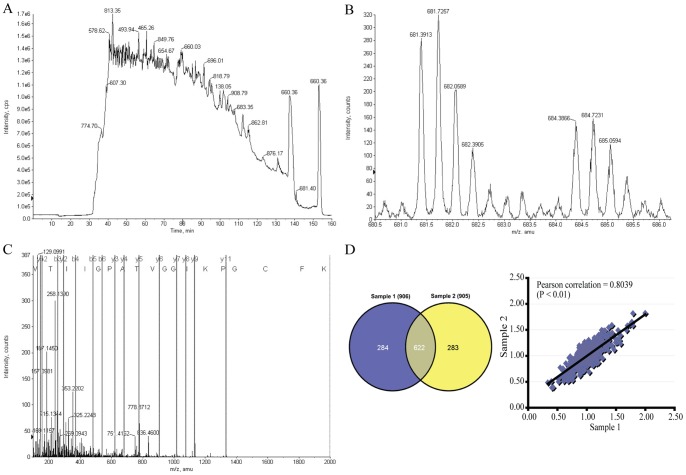
Protein samples from LX2/miR-27a and LX2/miR-neg were compared by cleavable isotope-coded affinity tag (cICAT)-based quantitative proteomic analysis - identification and quantitation of ATP-citrate synthase. (A) Total ion chromatogram (TIC) indicating cICAT-labeled peptides eluting from a reverse phase column. (B) Expanded MS spectrum view of a pair of peaks showing the differential expression between peptides labeled with the isotopically light and heavy cICAT reagent. (C) MS/MS spectrum analysis of the light-cICAT labeled triply charged peptide (681.4 *m/z*) showed in (B) led to identification of a peptide with sequence GVTIIGPATVGGIKPGCFK (ICAT-C(C)@17), unique to the ATP-citrate synthase (ACLY), a predicted target of miR-27a. The labels b and y designated the N- and C- terminal fragments, respectively, of the peptide produced by breakage at the peptide bond in the mass spectrometer. The number represents the number of N- or C- terminal residues present in the peptide fragment. (D) Venn diagram depicting the overlap of proteins identified in two independent cICAT experiments. Numbers in parentheses indicate the number of identified proteins for each sample. To examine the biological reproducibility, linear regression analyses were performed on H/L ratios (LX2/miR-27a/LX2/miR-neg) of two independent analyses. Pearson correlation coefficient between samples 1 and 2 was 0.8039, P<0.01.

Global protein expression profiles were compared between LX2/miR-27a and LX2/miR-neg stable transfectants by a cICAT-based quantitative proteomic approach (Figure 2A–C). Two biological replications were analyzed (Table S2). To estimate the analytical reproducibility of our proteomics study, linear regression analyses were performed on H/L ratios of duplicate analyses of samples 1 and 2 (Figure 2D). Pearson correlation coefficient for sample 1 and 2 was 0.8039 (P<0.01). Thus, the ratios of the two duplicate analyses were significantly positively correlated, indicating the good analytical reproducibility of the on-line 2D LC/MS/MS system. Thereby, spectral data from two duplicate analyses were merged and searched again to enhance the coverage of protein identification and to “average” the expression ratios of proteins identified in samples 1 and 2 (Table S3).

In the present study, 1267 non-redundant proteins were identified with unique accession numbers (score ≥1.3, i.e. confidence ≥95%), among which 1171 were quantified (Table S3). In the present study, based on the expression ratio of housekeeping proteins such as β-actin (ACTB, H/L = 1.0637) and tubulin β chain (TUBB, H/L = 1.0274), a differential protein expression ratio of 1.5 was selected as significant threshold [17], thus 149 (12.72%) proteins were differentially expressed. Of these 149 proteins, 74 were up-regulated (i.e. H/L ≥1.5) and 75 were down-regulated (i.e. H/L ≤0.6667), the number of up-regulated proteins was almost equal to that of down-regulated (Table S4). Compared with our previous study on HSCs activation [18], the extent of protein expression changes is relatively small in miR-27a overexpressed LX2, only 6 proteins increase up to 3-fold (i.e. H/L ≥3.0) and 2 proteins reduced below 3-fold (i.e. H/L ≤0.3333). The results also corroborated a recent finding that a single miRNA could regulate the production of hundreds of proteins, but the regulation was typically relatively mild [5].

### 3. Correlation between miR-27a target prediction and down-regulated proteins in LX2/miR-27a identified by cICAT

Next, we tried to figure out how miR-27a target prediction correlated with miR-27a down-regulated proteins in HSCs identified by cICAT-based proteomics analyses. TargetScan is one of the widely recognized databases for biological targets prediction of miRNAs [19]. By searching TargetScan Human Release 6.2 (http://www.targetscan.org/vert_61/), we found that only 2 out of the 75 down-regulated proteins were predicted targets of miR-27a, namely SMAD5 (mothers against decapentaplegic homolog 5) and ACLY (ATP-citrate synthase). SMAD5, a key component of TGF-beta signaling pathway, is an experimentally confirmed target of miR-27 [20]. ACLY is the primary enzyme responsible for the synthesis of cytosolic acetyl-CoA in many tissues and has a central role in de novo lipid synthesis. We further searched the predicted consequential pairing of miR-27a target region in the 3′ UTR of the remaining 73 down-regulated proteins in TargetScan Human Release 6.2. As shown in Table 1, 15 (20%) out of 75 down-regulated proteins could be potential targets of miR-27a, while the other 60 (80%) down-regulated proteins did not have consequential pairing of miR-27a target region in the 3′ UTR. Moreover, 74 proteins were even up-regulated in LX2/miR-27a stable transfectants. These findings suggested that the miRNA responsive proteins were not necessarily the predicted endogenous targets, they also reflected indirect effects. The underlying mechanisms deserve further investigation, as it has also been reported that miRNAs can even stimulate gene expression post transcriptionally by direct and indirect mechanisms [21].

**Table 1:** Predicted miR-27a Targets among Down-regulated Proteins in LX2/miR-27a Identified by cICAT.

Gene symbol	Accession	Predicted consequentialpairing of target region(top) and miRNA (bottom)		Seed match	Context score	Context scorepercentile	P_CT_ *	H/L
**ACLY**	NM_001096	Position 697–703 of ACLY 3' UTR	5′ …UGGAAAUGCAGAAAGCUGUGAAA…	7mer-1A	−0.13	73	0.67	0.6597
			||||||					
		hsa-miR-27a	3′ CGCCUUGAAUCGGUGACACUU					
								
**AP3D1**	NM_001077523	Position 187–193 of AP3D1 3′ UTR	5′ …UGACCAUCCUUUUUUACUGUGAC…	7mer-m8	−0.20	87	<0.1	0.5462
			||| |||||||					
		hsa-miR-27a	3′ CGCCUUGAAUCGGUGACACUU					
								
**ATP2A2**	NM_170665	Position 2249–2256 of ATP2A2 3′ UTR	5′ …AAAAAAAUCAGCCUUACUGUGAA…	8mer	>−0.03	2	<0.1	0.6095
			|||||||					
		hsa-miR-27a	3′ CGCCUUGAAUCGGUGACACUU					
								
**COPA**	NM_001098398	Position 1233–1239 of COPA 3′ UTR	5′ …UGAGGACCUAAACUGCUGUGAAA…	7mer-1A	−0.11	63	<0.1	0.6641
			||||||					
		hsa-miR-27a	3′ CGCCUUGAAUCGGUGACACUU					
								
**DYNLL2**	NM_080677	Position 535–541 of DYNLL2 3′ UTR	5′ …AGAAUAUUCCACUGAACUGUGAU…	7mer-m8	−0.12	71	0.34	0.4487
			|||||||					
		hsa-miR-27a	3′ CGCCUUGAAUCGGUGACACUU					
								
**FN1**	NM_002026	Position 431–437 of FN1 3′ UTR	5′ …AAGCAUGAUCUUGUU-ACUGUGAU…	7mer-m8	−0.22	89	<0.1	0.5669
			||| |||||||					
		hsa-miR-27a	3′ CGCCUUGAAUCGGUGACACUU					
		Position 742–748 of FN1 3′ UTR	5′ …CGGGGGAAAUAAUUCCUGUGAAU…	7mer-1A	−0.13	71	<0.1	
			||||||					
		hsa-miR-27a	3′ CGCCUUGAAUCGGUGACACUU					
								
**GNPNAT1**	NM_198066	Position 175–181 of GNPNAT1 3′ UTR	5′ …GGCUGGUGGGACAUGCUGUGAAU…	7mer-1A	−0.12	68	<0.1	0.5175
			||||||					
		hsa-miR-27a	3′ CGCCUUGAAUCGGUGACACUU					
		Position 668–674 of GNPNAT1 3′ UTR	5′ …UACCACUUGUCUUUUCUGUGAAU…	7mer-1A	−0.10	60	<0.1	
			|||| ||||||					
		hsa-miR-27a	3′ CGCCUUGAAUCGGU–GACACUU					
								
**H6PD**	NM_004285	Position 1513–1519 of H6PD 3′ UTR	5′ …GAGCAUAGGUUGGGGACUGUGAU…	7mer-m8	> −0.02	0	<0.1	0.5198
			|||||||					
		hsa-miR-27a	3′ CGCCUUGAAUCGGUGACACUU					
		Position 5755–5761 of H6PD 3′ UTR	5′ …UGUGCCGGAGUGGGAACUGUGAU…	7mer-m8	−0.02	27	<0.1	
			|||||||					
		hsa-miR-27a	3′ CGCCUUGAAUCGGUGACACUU					
								
**HSD17B12**	NM_016142	Position 1071–1078 of HSD17B12 3′ UTR	5′ …AAGAAAGAAUUCAAUACUGUGAA…	8mer	−0.33	97	<0.1	0.3966
			|||||||					
		hsa-miR-27a	3′ CGCCUUGAAUCGGUGACACUU					
								
**PAK2**	NM_002577	Position 2076–2082 of PAK2 3′ UTR	5′ …CAACGAGAUGAGAAGACUGUGAU…	7mer-m8	> −0.02	2	<0.1	0.5688
			|||||||					
		hsa-miR-27a	3′ CGCCUUGAAUCGGUGACACUU					
								
**PPM1B**	NM_001033557	Position 177–184 of PPM1B 3′ UTR	5′ …AUUAAACUUUAAAUGACUGUGAA…	8mer	−0.40	99	<0.1	0.4537
			||||| |||||||					
		hsa-miR-27a	3′ CGCCUUGAAUCGG–UGACACUU					
								
**RAB23**	NM_016277	Position 982–988 of RAB23 3′ UTR	5′ …GUCAUUCAGGAGGUCCUGUGAAG…	7mer-1A	−0.01	23	<0.1	0.6407
			||||||					
		hsa-miR-27a	3′ CGCCUUGAAUCGGUGACACUU					
								
**SEC61A1**	NM_013336	Position 197–204 of SEC61A1 3′ UTR	5′ …GCACUGGCAAAAAGAACUGUGAA…	8mer	−0.30	95	<0.1	0.5849
			|||||||					
		hsa-miR-27a	3′ CGCCUUGAAUCGGUGACACUU					
								
**SMAD5**	NM_001001419	Position 72–78 of SMAD5 3′ UTR	5′ …ACUUUGAGUACAGAUACUGUGAG…	7mer-m8	−0.20	87	0.75	0.6113
			|||||||					
		hsa-miR-27a	3′ CGCCUUGAAUCGGUGACACUU					
		Position 2427–2433 of SMAD5 3′ UTR	5′ …UUAUUGGUGUUUUCUACUGUGAG…	7mer-m8	−0.03	31	<0.1	
			|||||||					
		hsa-miR-27a	3′ CGCCUUGAAUCGGUGACACUU					
								
**SPTBN1**	NM_178313	Position 2130–2136 of SPTBN1 3′ UTR	5′ …UCAUUUGAUCAUAGCACUGUGAU…	7mer-m8	−0.16	81	<0.1	0.6351
			|||||||					
		hsa-miR-27a	3′ CGCCUUGAAUCGGUGACACUU					

* P_CT,_ the probability of conserved targeting.

### 4. Validation of proteomic findings by real-time RT-PCR

**Figure 3:**
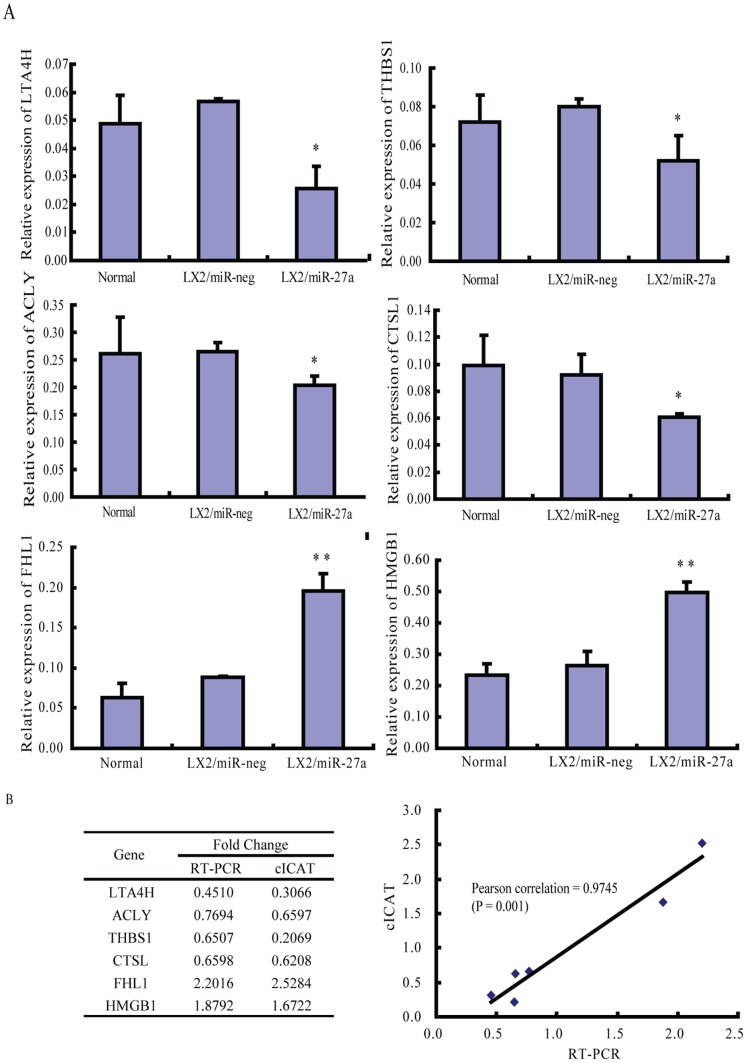
Validation of cICAT proteomic findings by real-time RT-PCR. (A) The expression of 6 genes encoding selected proteins in LX2/miR-27a stable transfectants. (B) Linear regression analysis of the fold change of protein and encoding gene in LX2/miR-27a detected by cICAT and RT-PCR respectively. ACLY, ATP-citrate synthase; LTA4H, leukotriene A4 hydrolase; CTSL1, cathepsin L1; THBS1, thrombospondin-1 precursor; FHL1, four and a half LIM domains 1; HMGB1, high-mobility group box 1. *P<0.05, **P<0.01 compared with LX2/miR-neg.

Six of the differentially expressed proteins identified in two replicate cICAT assays, ATP-citrate synthase (ACLY), leukotriene A4 hydrolase (LTA4H), cathepsin L1 (CTSL1), thrombospondin-1 precursor (THBS1), four and a half LIM domains 1 (FHL1) and high-mobility group box 1(HMGB1), were validated by real-time RT-PCR. The relationship between fold changes of protein detected by cICAT and fold changes of protein encoding gene detected by PCR was assessed by linear regression analysis. Pearson correlation coefficient for cICAT and real-time RT-PCR expression data was 0.9745 (P = 0.001). The PCR results confirmed the expression pattern observed in cICAT quantitative proteomics analysis (Figure 3).

### 5. Overall distribution of miR-27a regulated proteins in LX2 cells

**Figure 4:**
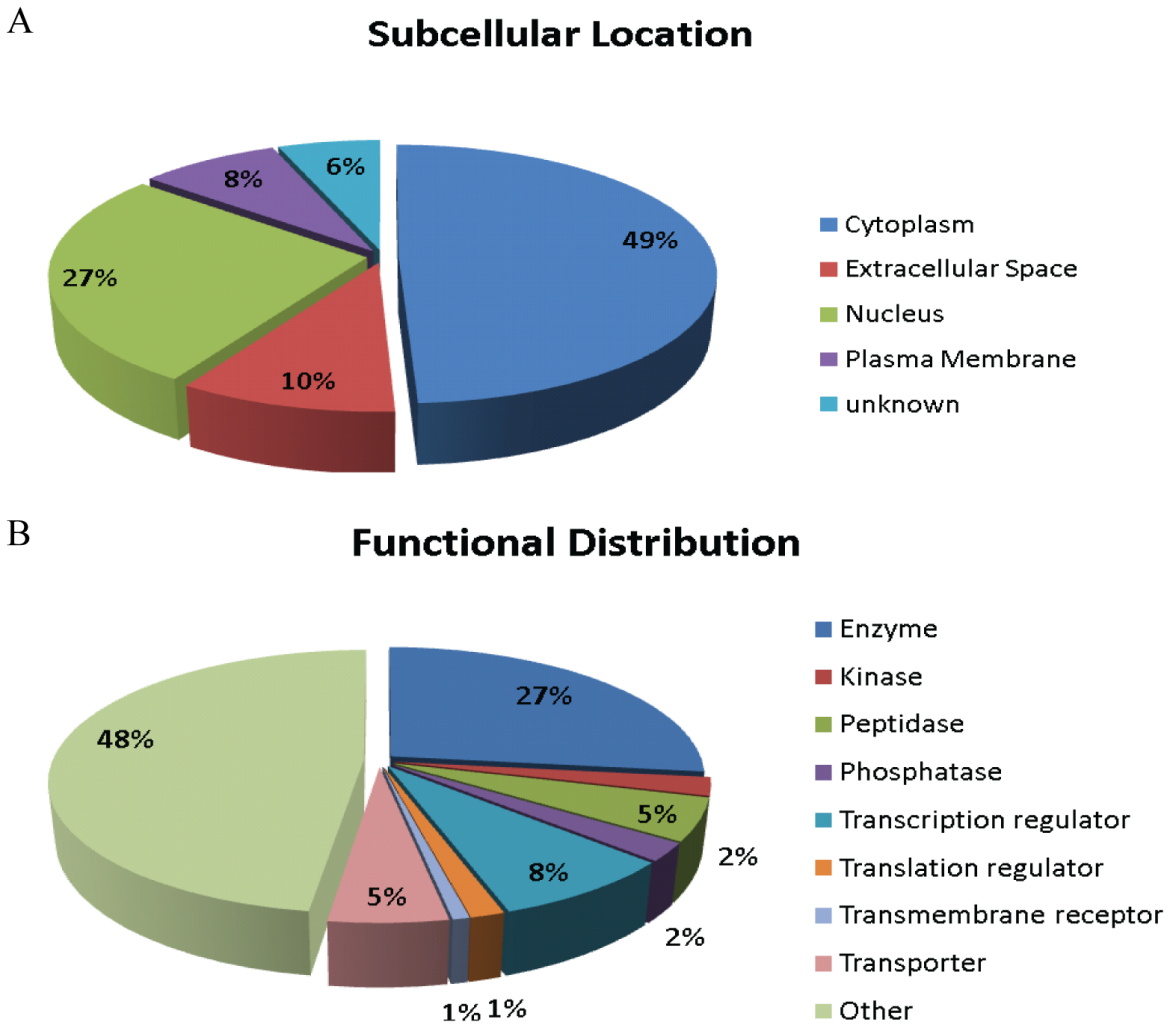
Overall distribution of miR-27a regulated proteins in LX2 cells. (A) Cell location and (B) Functional distribution of all the 134 differentially expressed proteins.

The subcellular location and bio-function of miR-27a regulated proteins in LX2 cells were categorized by using Protein Knowledgebase (UniprotKB) (Table S4). The subcellular localization of miR-27a regulated proteins is wide, including cytoplasm, nucleus, plasma membrane and extracellular space (Figure 4A). Enzymes, kinase, peptidase and phosphatase constituted the largest part of miR-27a regulated proteins in LX2 cells (49 out of 134 annotated differentially expressed proteins, 37%), followed by transcription regulator (11 out of 134, 8%). Therefore, by preferentially influencing the expression of enzymes and transcription regulators, miR-27a could perform its bio-function with high efficiency (Figure 4B).

### 6. Bio-functional analysis of differentially expressed proteins in LX2/miR-27a stable transfectants

GOfact was used to identify the enriched functional categories. The data of functional categorizing was inspiring, according to their molecular functions, most of the altered proteins could be well assigned into the categories involved in de novo lipid synthesis, cell proliferation, apoptosis, cell adhesion and migration, which were closely associated with the mechanisms participating in HSCs activation (Table 2, 3).

**Table 2:** Functional Categories of Down-regulated Proteins in LX2/miR-27a Compared with LX2/miR-neg (H/L ≤0.6667).

Functional Categories	Accession	Gene Symbol	Name	H/L	FunctionalCategories	Accession	Gene Symbol	Name	H/L
Lipid metabolism					Cell adhesionand mobility				
	IPI00021290.5	ACLY	ATP-citrate synthase	0.6597		IPI00394837.2	RAC1	ras-related C3 botulinum toxin substrate 1 isoform Rac1c	0.6298
	IPI00219077.4	LTA4H	Isoform 1 of Leukotriene A-4 hydrolase	0.3066		IPI00031008.1	TNC	Isoform 1 of Tenascin precursor	0.6217
	IPI00007676.3	HSD17B12	Estradiol 17-beta-dehydrogenase 12	0.3966		IPI00845263.1	FN1	fibronectin 1 isoform 2 preproprotein	0.5669
	IPI00022793.5	HADHB	Trifunctional enzyme subunit beta, mitochondrial precursor	0.4545		IPI00218803.2	FBLN1	Isoform B of Fibulin-1 precursor	0.4012
	IPI00169285.5	P76	Putative phospholipase B-like 2 precursor	0.6120		IPI00296099.6	THBS1	Thrombospondin-1 precursor	0.2069
**Glycolysis and TCA**						IPI00011285.1	CAPN1	Calpain-1 catalytic subunit	0.5367
	IPI00217143.3	SDHA	57 kDa protein	0.6594		IPI00844394.1	CYR61	42 kDa protein	0.5468
	IPI00790739.1	ACO2	Aconitase 2, mitochondrial	0.4723		IPI00872386.1	BCAR1	Breast cancer anti-estrogen resistance protein 1	0.5436
	IPI00291006.1	MDH2	Malate dehydrogenase, mitochondrial precursor	0.5272		IPI00009198.3	TFPI2	Tissue factor pathway inhibitor 2 precursor	0.4616
	IPI00607861.2	H6PD	GDH/6PGL endoplasmic bifunctional protein precursor	0.5198		IPI00007117.1	SERPINB2	Plasminogen activator inhibitor 2 precursor	0.5357
	IPI00643196.1	PFKP	Phosphofructokinase, platelet	0.5484	**Cytoskeleton**				
	IPI00418262.4	ALDOC	Fructose-bisphosphate aldolase C	0.5835		IPI00871932.1	SPTBN1	276 kDa protein	0.6351
**Cell growth related**						IPI00456969.1	DYNC1H1	Cytoplasmic dynein 1 heavy chain 1	0.6607
	IPI00869040.1	NUBP1	Isoform 2 of Nucleotide-binding protein 1	0.6392		IPI00062037.1	DYNLL2	Dynein light chain 2, cytoplasmic	0.4487
	IPI00419273.5	CUL4A	Isoform 1 of Cullin-4A	0.5050		IPI00146935.4	DNM1L	Isoform 1 of Dynamin-1-like protein	0.4586
	IPI00788802.1	TKT	Transketolase variant (Fragment)	0.6588	**Ubl conjugation pathway**				
**Transcription/translation regulator**						IPI00871372.1	HECTD1	HECT domain containing 1	0.3967
	IPI00025091.3	RPS11	40S ribosomal protein S11	0.6222		IPI00645078.1	UBA1	Ubiquitin-like modifier-activating enzyme 1	0.5802
	IPI00219156.7	RPL30	60S ribosomal protein L30	0.6370	**Miscellaneous**				
	IPI00738381.2	EEF1G	Elongation factor 1-gamma	0.6504		IPI00384428.3	BPHL	Isoform 1 of Valacyclovir hydrolase precursor	0.4093
	IPI00017730.1	SMAD5	Mothers against decapentaplegic homolog 5	0.6113		IPI00746782.1	MPST	3-mercaptopyruvate sulfurtransferase variant (Fragment)	0.4171
	IPI00215888.4	SRP72	Signal recognition particle 72 kDa protein	0.6129		IPI00026612.1	PPM1B	Isoform Beta-1 of Protein phosphatase 1B	0.4537
	IPI00376317.4	EDC4	Isoform 1 of Enhancer of mRNA-decapping protein 4	0.5609		IPI00019568.1	F2	Prothrombin precursor (Fragment)	0.5520
**Transport**						IPI00019903.1	CCDC44	Coiled-coil domain-containing protein 44	0.5392
	IPI00008034.1	RAB23	Ras-related protein Rab-23	0.6407		IPI00554521.2	FTH1	Ferritin heavy chain	0.6172
	IPI00791106.2	SCAMP4	Isoform 3 of Secretory carrier-associated membrane protein 4	0.6565		IPI00291136.4	COL6A1	Collagen alpha-1(VI) chain precursor	0.5397
	IPI00060287.3	C3orf31	MMP37-like protein, mitochondrial precursor	0.6380		IPI00872430.1	RPS8	25 kDa protein	0.5161
	IPI00029557.3	GRPEL1	GrpE protein homolog 1, mitochondrial precursor	0.6625		IPI00827508.2	RPL10A	25 kDa protein	0.5912
	IPI00646493.1	COPA	coatomer protein complex, subunit alpha isoform 1	0.6641		IPI00061525.3	GNPNAT1	Glucosamine 6-phosphate N-acetyltransferase	0.5175
	IPI00219078.5	ATP2A2	Isoform SERCA2B of Sarcoplasmic/endoplasmic reticulum calcium ATPase 2	0.6095		IPI00873294.1	BLMH	61 kDa protein	0.6072
	IPI00026530.4	LMAN1	Protein ERGIC-53 precursor	0.4662		IPI00289159.3	GLS	Isoform KGA of Glutaminase kidney isoform, mitochondrial precursor	0.6137
	IPI00178314.1	STXBP6	Isoform 1 of Syntaxin-binding protein 6	0.5278		IPI00219029.3	GOT1	Aspartate aminotransferase, cytoplasmic	0.6165
	IPI00411453.3	AP3D1	Isoform 1 of AP-3 complex subunit delta-1	0.5462		IPI00012887.1	CTSL1	Cathepsin L1 precursor	0.6208
	IPI00218466.6	SEC61A1	Isoform 1 of Protein transport protein Sec61 subunit alpha isoform 1	0.5849		IPI00022334.1	OAT	Ornithine aminotransferase, mitochondrial precursor	0.6457
	IPI00022881.1	CLTCL1	Isoform 1 of Clathrin heavy chain 2	0.5929		IPI00295386.7	CBR1	Carbonyl reductase [NADPH] 1	0.6148
	IPI00550382.2	SLC29A1	Equilibrative nucleoside transporter 1	0.5941		IPI00413986.2		Ribosomal protein L1	0.5311
	IPI00328181.1	TCIRG1	T-cell, immune regulator 1 isoform a	0.5663	**Hypothetical proteins**				
**Apoptosis**						IPI00738655.2	LOC653781	similar to protein expressed in prostate, ovary, testis, and placenta 2	0.6075
	IPI00010277.1	TNFRSF12A	Isoform 1 of Tumor necrosis factor receptor superfamily member 12A precursor	0.6016		IPI00788011.2	LOC728622	similar to S-phase kinase-associated protein 1A	0.5591
	IPI00419979.3	PAK2	Serine/threonine-protein kinase PAK 2	0.5688		IPI00888100.1	LOC390956	similar to peptidylprolyl isomerase A-like	0.5376
	IPI00847689.1	HTATIP2	HIV-1 Tat interactive protein 2, 30kDa isoform a	0.6114		IPI00847300.1		Similar to Voltage-dependent anion-selective channel protein 1	0.5335
						IPI00888597.1	LOC100129762	similar to KIAA0367	0.5103
						IPI00737530.1	LOC653888	similar to p41-Arc	0.4929

Proteins from LX2/miR-27a were labeled with heavy isotope (H) tagging and those from LX2/miR-neg were labeled with light isotope (L) tagging. Data were from two independent cICAT-based quantitative analyses.

**Table 3:** Functional Categories of Up-regulated Proteins in LX2/miR-27a Compared with LX2/miR-neg (H/L ≥1.5).

Functional Categories	Accession	Gene Symbol	Name	H/L	Functional Categories	Accession	Gene Symbol	Name	H/L
Lipid metabolism					Apoptosis				
	IPI00872459.2	PRKAA1	Uncharacterized protein PRKAA1	1.9474		IPI00893062.1	XRCC6	X-ray repair complementing defective repair in Chinese hamster cells 6	1.5110
**DNA replication and cell growth**						IPI00010882.3	DFFA	Isoform DFF45 of DNA fragmentation factor subunit alpha (Fragment)	2.0058
	IPI00163608.1	PARD3	Isoform 5 of Partitioning-defective 3 homolog	1.5964		IPI00006904.1	AVEN	Cell death regulator Aven	1.5283
	IPI00219420.3	SMC3	Structural maintenance of chromosomes protein 3	1.5081	**Cell adhesion and mobility**				
	IPI00791117.1	TK1	29 kDa protein	1.7692		IPI00010676.1	PLAUR	Isoform 1 of Urokinase plasminogen activator surface receptor precursor	1.5458
	IPI00465044.2	RCC2	Protein RCC2	1.7793	**Cytoskeleton**				
	IPI00419258.4	HMGB1	High mobility group protein B1	1.6722		IPI00220278.5	MYL9	Myosin regulatory light chain 2, smooth muscle isoform	1.5910
	IPI00031517.1	MCM6	DNA replication licensing factor MCM6	1.6907		IPI00328113.2	FBN1	Fibrillin-1 precursor	1.5611
	IPI00013679.1	DUT	Isoform DUT-M of Deoxyuridine 5′-triphosphate nucleotidohydrolase, mitochondrial precursor	1.6977		IPI00013991.1	TPM2	Isoform 1 of Tropomyosin beta chain	1.6519
	IPI00384967.3	ALDH1A3	Putative uncharacterized protein DKFZp686G1675 (Fragment)	1.8431		IPI00442894.3	TPM1	Tropomyosin alpha-1 chain	1.8151
	IPI00002135.1	TACC3	Transforming acidic coiled-coil-containing protein 3	1.6166		IPI00336047.5	MYO9B	Isoform Long of Myosin-IXb	2.3887
	IPI00014572.1	SPARC	SPARC precursor	1.7071		IPI00398735.3	CNN2	calponin 2 isoform b	1.6890
	IPI00034181.1	RBBP9	Isoform 1 of Retinoblastoma-binding protein 9	1.7084		IPI00844425.1	C3orf10	Isoform 2 of Probable protein BRICK1	2.0215
	IPI00014398.2	FHL1	Four and a half LIM domains 1 variant	2.5284		IPI00183002.6	PPP1R12A	Isoform 1 of Protein phosphatase 1 regulatory subunit 12A	1.9959
**Transcription/translation regulator**						IPI00478231.2	RHOA	Transforming protein RhoA precursor	1.5511
	IPI00011675.1	SP100	Isoform Sp100-HMG of Nuclear autoantigen Sp-100	1.5817	**Ubl conjugation pathway**				
	IPI00604620.3	NCL	NCL Isoform 1 of Nucleolin	1.6097		IPI00874175.1	UBE2G2	Ubiquitin carrier protein (Fragment)	1.8507
	IPI00647163.1	TCEAL4	Isoform 2 of Transcription elongation factor A protein-like 4	1.5207	**Miscellaneous**				
	IPI00219097.4	HMGB2	High mobility group protein B2	1.7124		IPI00163230.5	COPS6	COP9 signalosome complex subunit 6	6.9577
	IPI00853059.2	FUBP1	Isoform 2 of Far upstream element-binding protein 1	1.7293		IPI00477962.3	UAP1L1	Isoform 1 of UDP-N-acetylhexosamine pyrophosphorylase-like protein 1	2.0940
	IPI00167985.5	ZNF579	Zinc finger protein 579	1.8441		IPI00296141.3	DPP7	Dipeptidyl-peptidase 2 precursor	1.8415
	IPI00007941.4	HEXIM1	Protein HEXIM1	1.8459		IPI00026087.1	BANF1	Barrier-to-autointegration factor	1.6141
	IPI00028122.1	PSIP1	Isoform 1 of PC4 and SFRS1-interacting protein	1.9394		IPI00807702.1	TNIP1	NEF-associated factor 1	1.5713
	IPI00855957.2	KHSRP	Isoform 2 of Far upstream element-binding protein 2	2.0065		IPI00101968.3	DBNL	Isoform 3 of Drebrin-like protein	1.6175
	IPI00215801.1	RBM39	Isoform 2 of RNA-binding protein 39	2.0987		IPI00093057.6	CPOX	Coproporphyrinogen III oxidase, mitochondrial precursor	1.5958
	IPI00871695.1	DEK	48 kDa protein	4.8877		IPI00103925.2	IRGQ	Immunity-related GTPase family Q protein	1.5803
	IPI00024662.1	CBX5	Chromobox protein homolog 5	1.8359		IPI00894202.1	C2orf30	chromosome 2 open reading frame 30 isoform 2	1.5903
	IPI00297579.4	CBX3	Chromobox protein homolog 3	1.7487		IPI00550308.1	RBM12	RNA-binding protein 12	1.5255
	IPI00021417.3	SART1	U4/U6.U5 tri-snRNP-associated protein 1	1.5333		IPI00031622.3	CHCHD6	Coiled-coil-helix-coiled-coil-helix domain-containing protein 6	3.5705
	IPI00555857.1	SFRS5	CS0DF038YO05 variant (Fragment)	1.7597		IPI00178750.3	NIP30	NEFA-interacting nuclear protein NIP30	2.2462
	IPI00026957.1	WBP4	WW domain-binding protein 4	1.7331		IPI00304922.1	LSMD1	Isoform 1 of LSM domain-containing protein 1	12.1912
	IPI00215884.4	SFRS1	Isoform ASF-1 of Splicing factor, arginine/serine-rich 1	1.5994		IPI00396321.1	LRRC59	Leucine-rich repeat-containing protein 59	1.7094
	IPI00290461.3	EIF3J	Eukaryotic translation initiation factor 3 subunit J	1.5853		IPI00297263.6	HEG1	Isoform 1 of Protein HEG homolog 1 precursor	1.9231
	IPI00552639.2	EIF4G1	Isoform 1 of Eukaryotic translation initiation factor 4 gamma 1	1.6356		IPI00419836.1	DCBLD2	Isoform 1 of Discoidin, CUB and LCCL domain-containing protein 2 precursor	1.8740
**Transport**					**Hypothetical proteins**				
	IPI00848342.1	LTF	Lactotransferrin precursor	1.6590		IPI00006932.3	LUC7L2	Isoform 1 of Putative RNA-binding protein Luc7-like 2	1.5778
	IPI00303402.7	RNUXA	RNA U small nuclear RNA export adapter protein	1.5796		IPI00333014.3	C13orf3	Isoform 1 of Uncharacterized protein C13orf3	1.6993
	IPI00449201.2	ATG3	Isoform 2 of Autophagy-related protein 3	1.5491		IPI00013832.3	GATC	GatC-like protein	1.5144
	IPI00871988.1	SFXN3	Uncharacterized protein SFXN3	1.6101		IPI00795769.1		52 kDa protein	2.0541
	IPI00641384.2	SEC16A	SEC16 homolog A	3.0693		IPI00472879.3		Novel protein similar to Pre-B cell enhancing factor	1.5245
	IPI00872163.1	ATP2A1	Similar to ATPase, Ca++ transporting, cardiac muscle, fast twitch 1	3.2500					

Proteins from LX2/miR-27a were labeled with heavy isotope (H) tagging and those from LX2/miR-neg were labeled with light isotope (L) tagging. Data were from two independent cICAT-based quantitative analyses.

**Figure 5:**
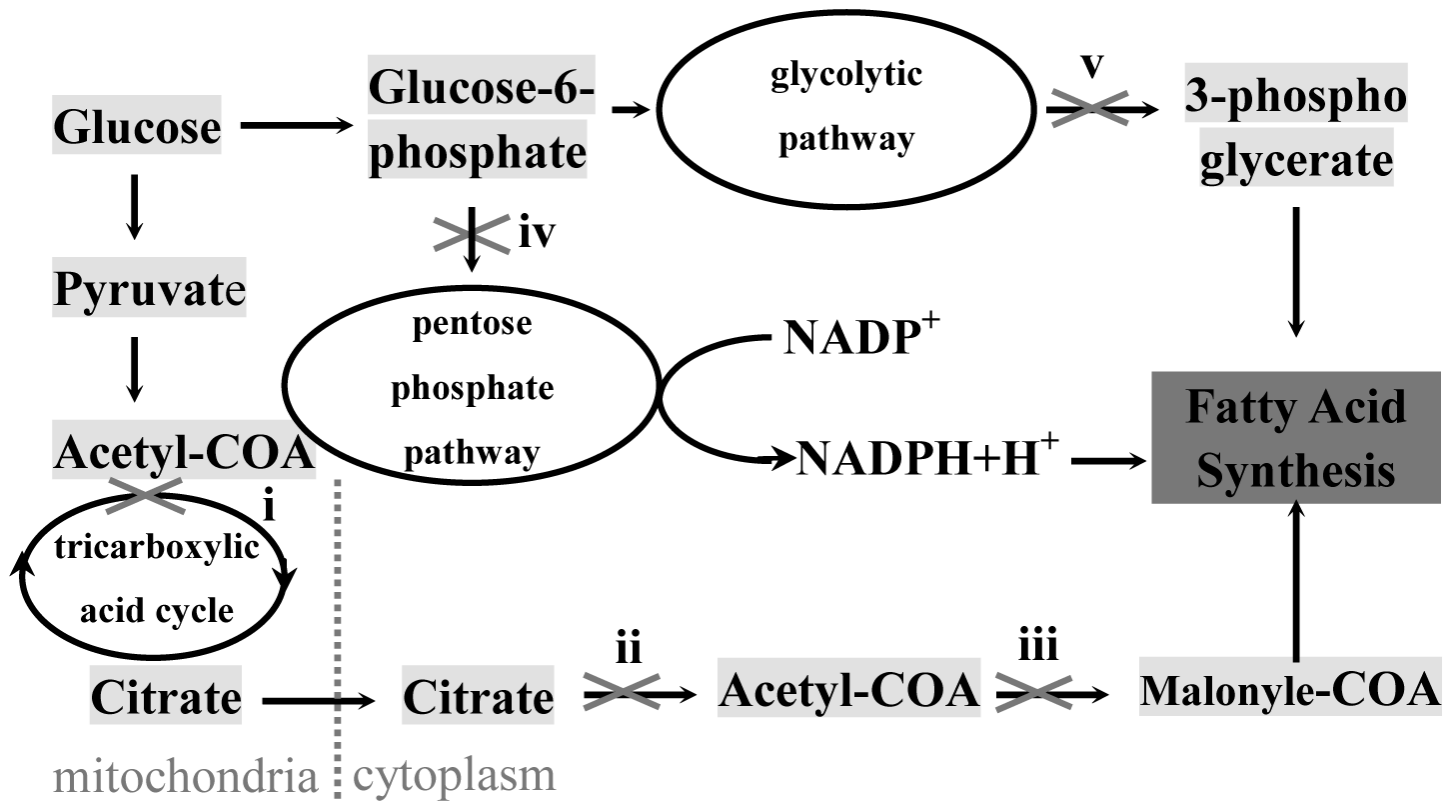
Altered proteins that are involved in metabolism processes related to de novo lipid synthesis: aconitase 2 (ACO2) and malate dehydrogenase (MDH2), which participate in tricarboxylic acid cycle (TAC) (i) decreased; ATP-citrate synthase (ACLY), the primary enzyme responsible for the synthesis of cytosolic acetyl-CoA (ii) decreased; 5′-AMP-activated protein kinase catalytic subunit alpha-1 (PRKAA1) that repress the synthesis of malonyl-CoA (iii) by phosphorylation of acetyl-CoA carboxylase increased; glucose 1-dehydrogenase/6-phosphogluconolactonase (H6PD), the rate-limiting enzyme in pentose phosphate pathway (PPP) (iv) decreased; 6-phosphofructokinase type C (PFKP) that acts as the rate-limiting enzyme, fructose-bisphosphate aldolase C (ALDOC), which are involved in glycolytic pathway(v) decreased.

A large number of the down-regulated proteins were involved in de novo lipid synthesis (Figure 5), among which three groups were most concerned: (1) aconitase (ACO2), malate dehydrogenase (MDH2), and ATP-citrate synthase (ACLY), which are important enzymes participating in tricarboxylic acid cycle and favor the production of acetyl-CoA; (2) glucose 1-dehydrogenase/6-phosphogluconolactonase (H6PD), the rate-limiting enzyme for pentose phosphate pathway that supplies NADPH; (3) 6-phosphofructokinase type C (PFKP) and fructose-bisphosphate aldolase C (ALDOC), are involved in glycolytic pathway that provides glycerol-3-phosphate, and the former is a rate-limiting enzyme (Table 2). Acetyl-CoA, NADPH and glycerol-3-phosphate are all required in de novo lipid synthesis. On the other hand, one negative regulator of lipid synthesis called 5′-AMP-activated protein kinase catalytic subunit alpha-1 (PRKAA1) was significantly up-regulated(Table 3). By phosphorylation, PRKAA1 can inactivate acetyl-CoA carboxylase that catalyzes the rate-limiting reaction in the biosynthesis of long-chain fatty acids [22], [23]. So miR-27a may affect HSCs fat accumulation by directly regulating a group of genes that are involved in the biosynthesis of triglyceride.

Proteins involved in cell adhesion and mobility constituted another major group of down-regulated proteins (10 out 75), including Tenascin (TNC) [24], fibronectin 1 (FN1) [25] and Fibulin-1 (FBLN1) [26], which correlated with reduced adhesion and increased migration of miR-27a stable transfectants (Figure 1D).

Over expression of miR-27a also up-regulated a group of factors that favorite proliferation of HSCs. Twelve out of 74 up-regulated proteins were DNA replication and growth-related, and 19 proteins were important transcription/translation regulators, e.g. DNA replication licensing factor MCM6 (MCM6), transcription elongation factor A protein-like 4 (TCEAL4), eukaryotic translation initiation factor 3 subunit J (EIF3J), eukaryotic translation initiation factor 4 gamma 1 (EIF4G1), retinoblastoma-binding protein 9 (RBBP9) [27] and FHL1 [28].

The present proteomic study not only provided the possible mechanism underlying the previously reported miR-27 function in HSCs, but also casted new light on a novel role of miR-27a in myogenesis, which was consistent with the myofibroblast trans-differentiation during HSCs activation. In 9 up-regulated cytoskeleton related proteins, 4 are structural constituents of muscle, including tropomyosin alpha-1 chain (TPM1), tropomyosin beta chain (TPM2), myosin-IXb (MYO9B) and myosin regulatory light chain 2 (MYL9); 4 are in regulation of actomyosin structure and function, including protein phosphatase 1 regulatory subunit 12A (PPP1R12A) [29]; calponin 2 (CNN2) [30]; transforming protein RhoA (RHOA) [31] and FHL1 [32]. The up-regulation of TPM1, MYO9B and MYL9 by miR-27a in LX2 cells was further validated by RT-PCR (Figure S1). In a previous study, it has also been evidenced that miR-27a can up-regulate cardiac myosin heavy chain (MHC) gene (β-MHC) expression via thyroid hormone signaling [33]. And miR-27a has also been reported to be able to influence muscle stem cell behavior [34]. It is the first time for us to recognize a novel role of miR-27a in promoting myogenic tans-differentiation in HSCs. The finding also suggested similar bio-functions of the same miRNA in different types of tissues or cells. However, further effort is needed to determine the role of miR-27a in myogenic trans-differentiation of activated HSCs.

### 7. The biological significance of miR-27a regulated protein in HSCs

**Figure 6:**
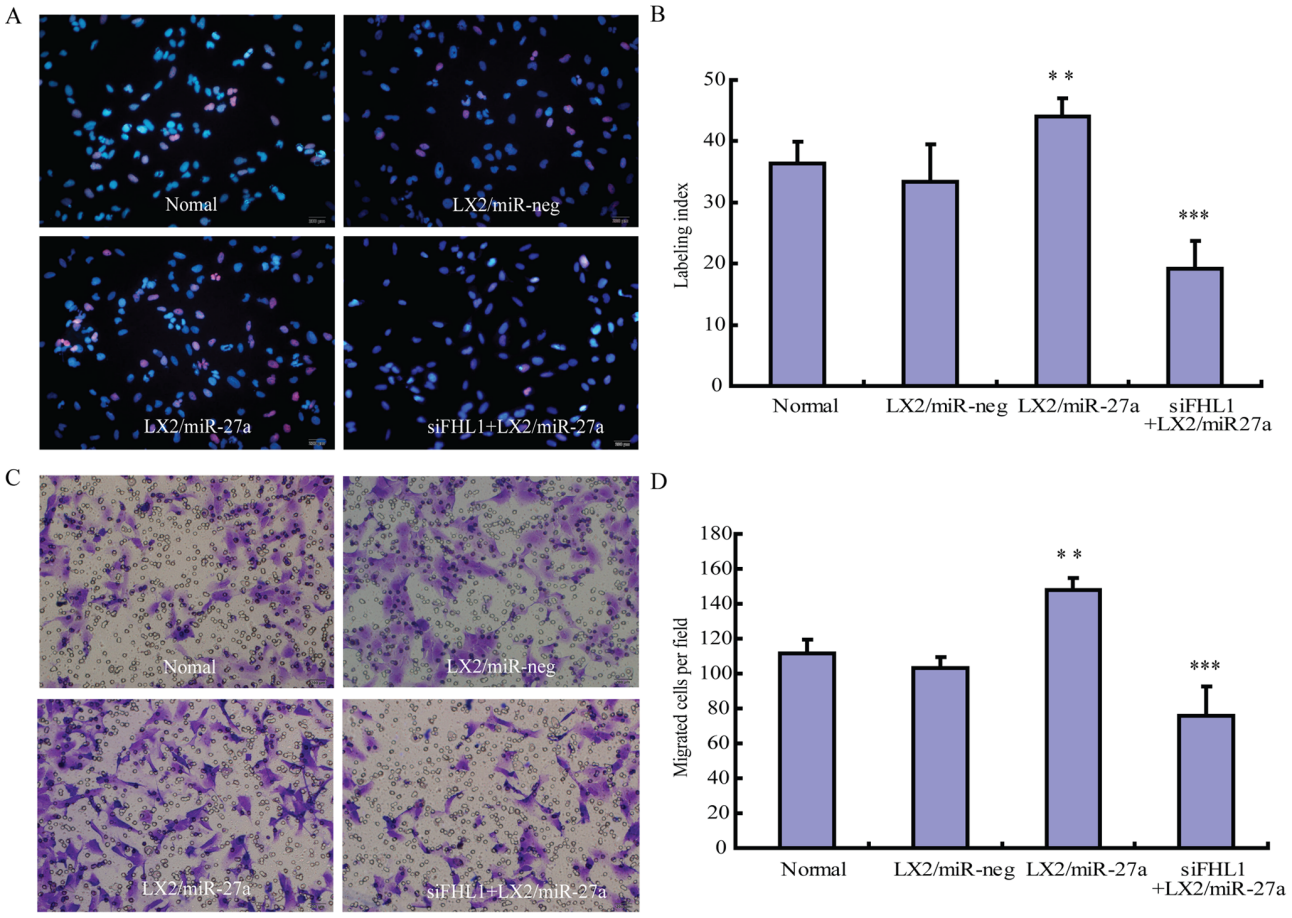
Involvement of FLH1 in miR-27a related HSCs proliferation and migration. Knockdown of FLH1 suppressed cell proliferation in LX2/miR-27a transfectants. (A) EdU cell proliferation assay. EdU was detected by Apollo 567 fluorescent dye (red) and nuclei were counterstained with Hoechst 33342 (blue) (original magnification ×200). (B) Statistical results of three independent experiments. The results are expressed as the labeling index according to the following formula: number of EdU-positive nuclei x 100/number of total nuclei. FHL1 was required for increased migration in LX2/miR-27a transfectants. (C) Migration assays. LX2/miR-27a transfectants were plated on 8-lm pore size Transwell inserts for 16 hours. The number of migrated cells was counted manually (original magnification ×200). (D) The statistical results of three independent experiments. Each image is a representative of three independent experiments. ***P<0.001, **P<0.01 compared with LX2/miR-neg.

In order to validate the biological significance of miR-27a regulated proteins identified by cICAT proteomic strategy, the function of FHL1, one of the highest increased proteins which not only related to cell growth [28] but also played a crucial role in embryonic skeletal muscle myogenesis [32], was evaluated in miR-27a transfectants. Three different siRNA targeting FHL1 were compared. The one possessed the highest knockdown efficiency (Figure S2) was used in the following experiment. Our data showed that FHL1 involved in miR-27a related HSCs proliferation and migration, knockdown of FHL1 significantly inhibited the proliferation and migration of LX2/miR-27a transfectants (Figure 6). Interestingly, in a recent study based on 2-dimensional polyacrylamide gel electrophoresis (2D-PAGE) proteomic approach, FHL-1 was identified as one of the most prominently up-regulated proteins in pulmonary hypertension mouse model, and a similar effects of FHL-1 on promoting pulmonary arterial smooth muscle cell migration and proliferation has also been evidenced [35].

## Conclusions

The data of present study indicated that miR-27a influenced the activation of HSCs by affecting several groups of proteins. These results not only explained our previous finding that over-expression of miR-27a promoted HSC activation with reduced cytoplasmic lipid drops and increased cell proliferation [8], but also revealed a novel role of miR-27a in promoting the myogenic trans-differentiation of activated HSC into myofibroblast. The pattern of miR-27a regulation on protein expression might well reflect the emerging picture of miRNA regulation in animals is far richer and more complex than the crisp linear pathways [1]. Our study also validated proteomic strategy as a promising tool for functional study of miRNA. In the future, it will be interesting to uncover the mechanisms underlying the regulation of miR-27a on these functionally related genes.

## Supporting Information

Figure S1 **Validation of myogenesis related genes found by cICAT proteomic analyses.** The expression of TPM1, MYO9B and MYL9 encoding mRNA was evaluated by RT-PCR in LX2/miR-27a stable transfectants. *P<0.05, compared with LX2/miR-neg.
(TIF)

Figure S2 **Knockdown efficiency of FHL1 siRNA, LX2 cells were transfected with FHL1 specific siRNA or with NTC siRNA, after 48 hours, their mRNA levels were determined by quantitative polymerase chain reaction.** GAPDH was used as housekeeping gene. NTC, non-targeting control siRNA transfected cells. **P<0.01 compared with NTC.
(TIF)

Table S1 **Primer Sets for Real-time PCR.** *Sense primers for mature miR-27a were provided here, anti-sense primer was provided by Invitrogen as Universal q-PCR Primer.
(DOC)

Table S2 **Protein List of 2 Independent 2D nano-LC-MS/MS Analysis of LX2/miR-27a and LX2/miR-neg.**
(XLS)

Table S3 **List of Proteins Identified and Quantified in LX2/miR-27a and LX2/miR-neg.**
(XLS)

Table S4 **List of Proteins Up-or Down-regulated in LX2/miR-27a Compared with LX2/miR-neg.**
(XLS)

## References

[1] Bartel DP (2009) MicroRNAs: target recognition and regulatory functions. Cell 136: 215–33. 19167326 10.1016/j.cell.2009.01.002 PMC3794896

[2] Griffiths-Jones S, Saini HK, van Dongen S, Enright AJ (2008) miRBase: tools for microRNA genomics. Nucleic Acids Res 36: D154–8. 17991681 10.1093/nar/gkm952 PMC2238936

[3] Friedman RC, Farh KK, Burge CB, Bartel DP (2009) Most mammalian mRNAs are conserved targets of microRNAs. Genome Res 19: 92–105. 18955434 10.1101/gr.082701.108 PMC2612969

[4] Thomas M, Lieberman J, Lal A (2010) Desperately seeking microRNA targets. Nat Struct Mol Biol 17: 1169–74. 20924405 10.1038/nsmb.1921

[5] Selbach M, Schwanhausser B, Thierfelder N, Fang Z, Khanin R, et al (2008) Widespread changes in protein synthesis induced by microRNAs. Nature 455: 58–63. 18668040 10.1038/nature07228

[6] Huang TC, Pinto SM, Pandey A (2013) Proteomics for understanding miRNA biology. Proteomics 13: 558–67. 23125164 10.1002/pmic.201200339 PMC3715049

[7] Shiio Y, Aebersold R (2006) Quantitative proteome analysis using isotope-coded affinity tags and mass spectrometry. Nat Protoc 1: 139–45. 17406225 10.1038/nprot.2006.22

[8] Ji J, Zhang J, Huang G, Qian J, Wang X, et al (2009) Over-expressed microRNA-27a and 27b influence fat accumulation and cell proliferation during rat hepatic stellate cell activation. FEBS Lett 583: 759–66. 19185571 10.1016/j.febslet.2009.01.034

[9] Wang T, Li M, Guan J, Li P, Wang H, et al (2011) MicroRNAs miR-27a and miR-143 Regulate Porcine Adipocyte Lipid Metabolism. Int J Mol Sci 12: 7950–9. 22174642 10.3390/ijms12117950 PMC3233448

[10] Vickers KC, Shoucri BM, Levin MG, Wu H, Pearson DS, et al (2013) MicroRNA-27b is a regulatory hub in lipid metabolism and is altered in dyslipidemia. Hepatology 57: 533–42. 22777896 10.1002/hep.25846 PMC3470747

[11] Xu W, Liu M, Peng X, Zhou P, Zhou J, et al (2013) miR-24–3p and miR-27a-3p promote cell proliferation in glioma cells via cooperative regulation of MXI1. Int J Oncol 42: 757–66. 23254855 10.3892/ijo.2012.1742

[12] Guttilla IK, White BA (2009) Coordinate regulation of FOXO1 by miR-27a, miR-96, and miR-182 in breast cancer cells. J Biol Chem 284: 23204–16. 19574223 10.1074/jbc.M109.031427 PMC2749094

[13] Acunzo M, Romano G, Palmieri D, Lagana A, Garofalo M, et al (2013) Cross-talk between MET and EGFR in non-small cell lung cancer involves miR-27a and Sprouty2. Proc Natl Acad Sci U S A 110: 8573–8. 23650389 10.1073/pnas.1302107110 PMC3666747

[14] Xu L, Hui AY, Albanis E, Arthur MJ, O’Byrne SM, et al (2005) Human hepatic stellate cell lines, LX-1 and LX-2: new tools for analysis of hepatic fibrosis. Gut 54: 142–51. 15591520 10.1136/gut.2004.042127 PMC1774377

[15] Dong L, Jianqi L, Shuguang O, Jian W, Xiaojie X, et al (2005) An Integrated Strategy for Functional Analysis in Large-scale Proteomic Research by Gene Ontology. Progress in Biochemistry and Biophysics 32: 1026–1029.

[16] Dong L, Jianqi L, Shuguang O, Songfeng W, Jian W, et al (2005) An integrated strategy for functional analysis in large scale proteomic research by gene ontology. Molecular & Cellular Proteomics. 4: S34–S34.

[17] Baek D, Villen J, Shin C, Camargo FD, Gygi SP, et al (2008) The impact of microRNAs on protein output. Nature 455: 64–71. 18668037 10.1038/nature07242 PMC2745094

[18] Ji J, Yu F, Ji Q, Li Z, Wang K, et al (2012) Comparative proteomic analysis of rat hepatic stellate cell activation: a comprehensive view and suppressed immune response. Hepatology 56: 332–49. 22331624 10.1002/hep.25650

[19] Lewis BP, Burge CB, Bartel DP (2005) Conserved seed pairing, often flanked by adenosines, indicates that thousands of human genes are microRNA targets. Cell 120: 15–20. 15652477 10.1016/j.cell.2004.12.035

[20] Rogler CE, Levoci L, Ader T, Massimi A, Tchaikovskaya T, et al (2009) MicroRNA-23b cluster microRNAs regulate transforming growth factor-beta/bone morphogenetic protein signaling and liver stem cell differentiation by targeting Smads. Hepatology 50: 575–84. 19582816 10.1002/hep.22982

[21] Ma F, Liu X, Li D, Wang P, Li N, et al (2010) MicroRNA-466l upregulates IL-10 expression in TLR-triggered macrophages by antagonizing RNA-binding protein tristetraprolin-mediated IL-10 mRNA degradation. J Immunol 184: 6053–9. 20410487 10.4049/jimmunol.0902308

[22] Carlson CA, Kim KH (1973) Regulation of hepatic acetyl coenzyme A carboxylase by phosphorylation and dephosphorylation. J Biol Chem 248: 378–80. 4692841

[23] Towler MC, Hardie DG (2007) AMP-activated protein kinase in metabolic control and insulin signaling. Circ Res 100: 328–41. 17307971 10.1161/01.RES.0000256090.42690.05

[24] Mackie EJ, Tucker RP, Halfter W, Chiquet-Ehrismann R, Epperlein HH (1988) The distribution of tenascin coincides with pathways of neural crest cell migration. Development 102: 237–50. 2458221 10.1242/dev.102.1.237

[25] Akiyama SK, Yamada SS, Chen WT, Yamada KM (1989) Analysis of fibronectin receptor function with monoclonal antibodies: roles in cell adhesion, migration, matrix assembly, and cytoskeletal organization. J Cell Biol 109: 863–75. 2527241 10.1083/jcb.109.2.863 PMC2115712

[26] Timpl R, Sasaki T, Kostka G, Chu ML (2003) Fibulins: a versatile family of extracellular matrix proteins. Nat Rev Mol Cell Biol 4: 479–89. 12778127 10.1038/nrm1130

[27] Shields DJ, Niessen S, Murphy EA, Mielgo A, Desgrosellier JS, et al (2010) RBBP9: a tumor-associated serine hydrolase activity required for pancreatic neoplasia. Proc Natl Acad Sci U S A 107: 2189–94. 20080647 10.1073/pnas.0911646107 PMC2836678

[28] Schawalder SB, Kabani M, Howald I, Choudhury U, Werner M, et al (2004) Growth-regulated recruitment of the essential yeast ribosomal protein gene activator Ifh1. Nature 432: 1058–61. 15616569 10.1038/nature03200

[29] Vicente-Manzanares M, Ma X, Adelstein RS, Horwitz AR (2009) Non-muscle myosin II takes centre stage in cell adhesion and migration. Nat Rev Mol Cell Biol 10: 778–90. 19851336 10.1038/nrm2786 PMC2834236

[30] Winder SJ, Allen BG, Clement-Chomienne O, Walsh MP (1998) Regulation of smooth muscle actin-myosin interaction and force by calponin. Acta Physiol Scand 164: 415–26. 9887965 10.1111/j.1365-201x.1998.tb10697.x

[31] Wei L, Zhou W, Croissant JD, Johansen FE, Prywes R, et al (1998) RhoA signaling via serum response factor plays an obligatory role in myogenic differentiation. J Biol Chem 273: 30287–94. 9804789 10.1074/jbc.273.46.30287

[32] Arber S, Halder G, Caroni P (1994) Muscle LIM protein, a novel essential regulator of myogenesis, promotes myogenic differentiation. Cell 79: 221–31. 7954791 10.1016/0092-8674(94)90192-9

[33] Nishi H, Ono K, Horie T, Nagao K, Kinoshita M, et al (2011) MicroRNA-27a regulates beta cardiac myosin heavy chain gene expression by targeting thyroid hormone receptor beta1 in neonatal rat ventricular myocytes. Mol Cell Biol 31: 744–55. 21149577 10.1128/MCB.00581-10 PMC3028640

[34] Crist CG, Montarras D, Pallafacchina G, Rocancourt D, Cumano A, et al (2009) Muscle stem cell behavior is modified by microRNA-27 regulation of Pax3 expression. Proc Natl Acad Sci U S A 106: 13383–7. 19666532 10.1073/pnas.0900210106 PMC2726381

[35] Kwapiszewska G, Wygrecka M, Marsh LM, Schmitt S, Trosser R, et al (2008) Fhl-1, a new key protein in pulmonary hypertension. Circulation 118: 1183–94. 18725486 10.1161/CIRCULATIONAHA.107.761916

