# Correction: Functional Study of miR-27a in Human Hepatic Stellate Cells by Proteomic Analysis: Comprehensive View and a Role in Myogenic Tans-Differentiation

**DOI:** 10.1371/journal.pone.0125950

**Published:** 2015-05-04

**Authors:** The PLOS ONE Staff

There is an error in affiliation 1 for author Yuhua Ji. Affiliation 1 should be: Key Laboratory of Neuroregeneration, Nantong University, Nantong, China. The publisher apologizes for the error.
