# Supplementary figures and images for: Functional Study of miR-27a in Human Hepatic Stellate Cells by Proteomic Analysis: Comprehensive View and a Role in Myogenic Tans-Differentiation
Source: PLoS One. 2014 Sep 29;9(9):e108351. doi: 10.1371/journal.pone.0108351 (PMC4180938; doi:10.1371/journal.pone.0108351)

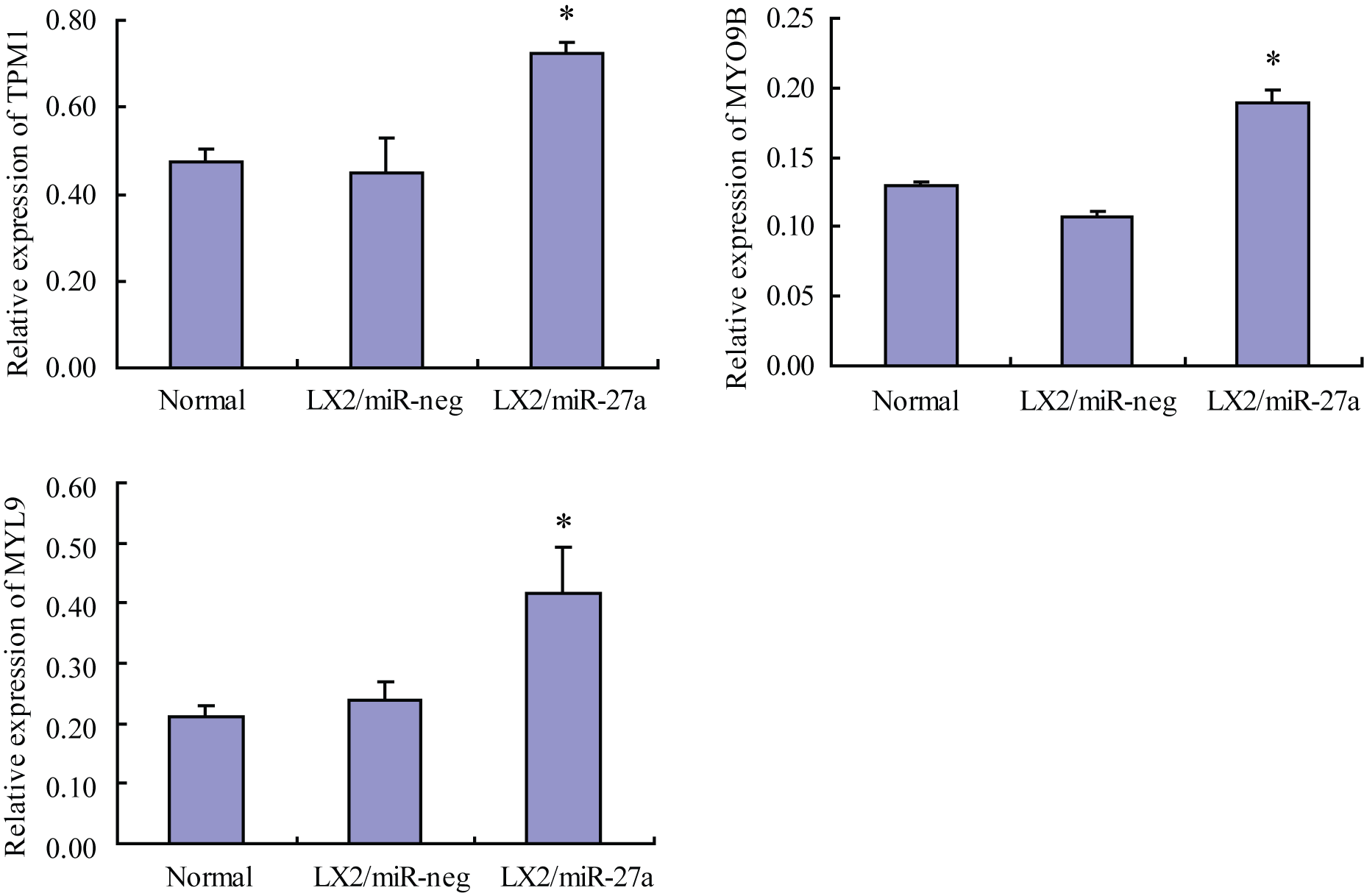

Supplement: Figure S1 — Validation of myogenesis related genes found by cICAT proteomic analyses. The expression of TPM1, MYO9B and MYL9 encoding mRNA was evaluated by RT-PCR in LX2/miR-27a stable transfectants. *P<0.05, compared with LX2/miR-neg. (TIF) [file pone.0108351.s001.tif]

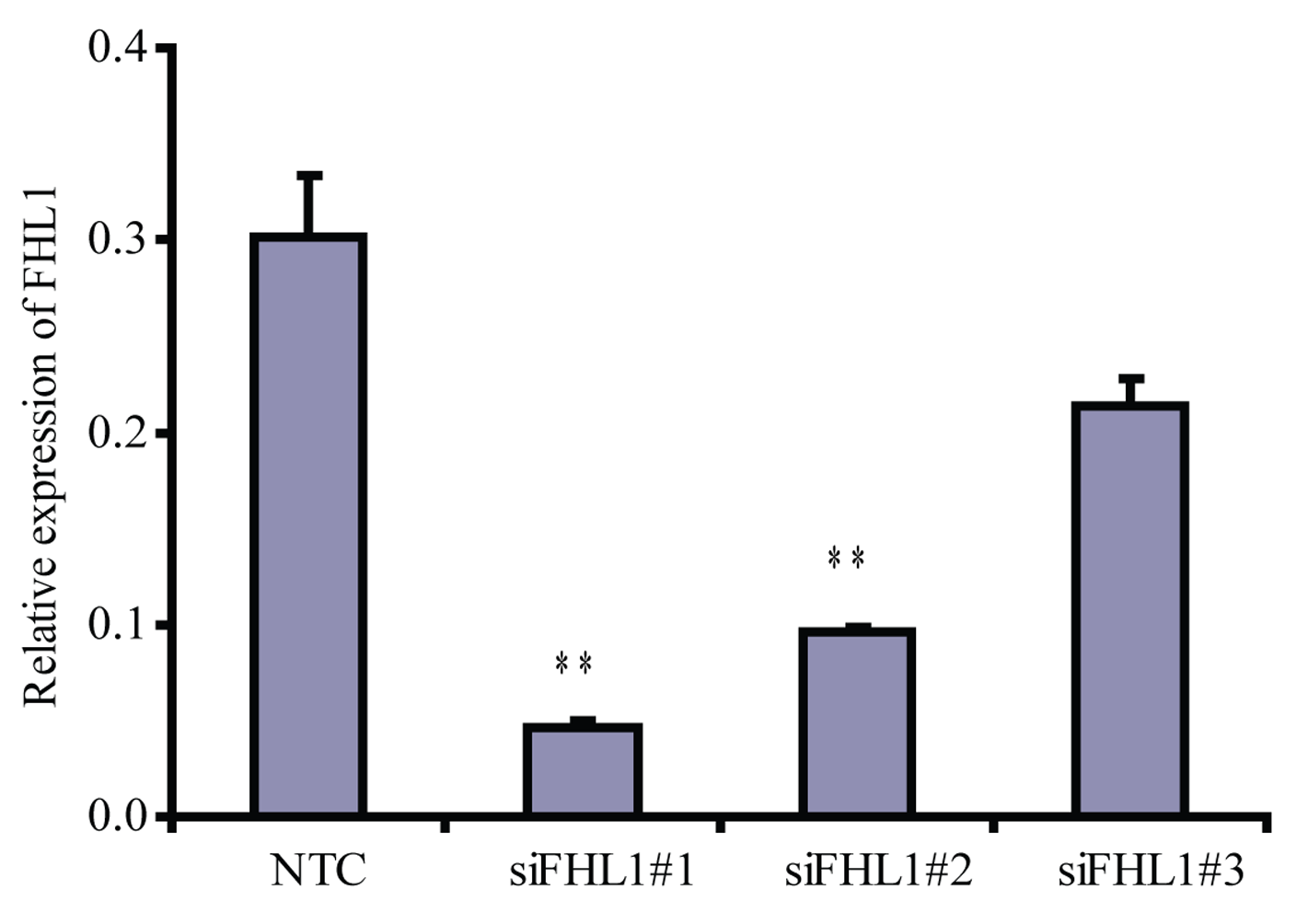

Supplement: Figure S2 — Knockdown efficiency of FHL1 siRNA, LX2 cells were transfected with FHL1 specific siRNA or with NTC siRNA, after 48 hours, their mRNA levels were determined by quantitative polymerase chain reaction. GAPDH was used as housekeeping gene. NTC, non-targeting control siRNA transfected cells. **P<0.01 compared with NTC. (TIF) [file pone.0108351.s002.tif]
