# Supplementary material for: Functional Study of miR-27a in Human Hepatic Stellate Cells by Proteomic Analysis: Comprehensive View and a Role in Myogenic Tans-Differentiation
Source: PLoS One. 2014 Sep 29;9(9):e108351. doi: 10.1371/journal.pone.0108351 (PMC4180938; doi:10.1371/journal.pone.0108351)
Supplement: Table S1 — Primer Sets for Real-time PCR. *Sense primers for mature miR-27a were provided here, anti-sense primer was provided by Invitrogen as Universal q-PCR Primer. (DOC) [file pone.0108351.s003.doc]

**Table S1 Primer Sets for Real-time PCR**

| **Gene** | **Sense** | **Anti-sense** | **Product Size (bp)** |
| --- | --- | --- | --- |
| **ACLY** | 5' TGATGGGAGAAGTCGGGAAGA 3' | 5' TGTGGCGGCTGAAGAGGGT 3' | 325 |
| **LTA4H** | 5' GGCAGGCGACAAGTCACTCT 3' | 5' CCAAACAATCGTCCGCAAA 3' | 153 |
| **CTSL** | 5' TGAATGAAGAAGGATGGAGGAG 3' | 5' AAAGCCCAACAAGAACCACA 3' | 298 |
| **THBS1** | 5' ACCGACTTCCGCCGATTC 3' | 5' TCTGGACGAGTTCTTTACCCTGAT 3' | 103 |
| **HMGB1** | 5' TATGGCAAAAGCGGACAAG 3' | 5' TTCCACATCTCTCCCAGTTTC 3' | 216 |
| **FHL1** | 5' GGAGATAAAACCCCCACTGA 3' | 5' CAGAAGGAAGGGCAGAGTTC 3' | 183 |
| **TPM1** | 5' AAGAGTTGGATCGTGCCCAG 3' | 5' CCTGAGCCTCCAGTGACTTC 3' | 342 |
| **MYO9B** | 5' AGCCAGTGAAGCAGAGCAAA 3' | 5' CCCTTCCTCCCGTAGGTGTA 3' | 241 |
| **MYL9** | 5' ACCCACCAGAAGCCAAGATG 3' | 5' ATGGATGAAACCCAGCGAGG 3' | 212 |
| **GAPDH** | 5' AGGTCATCCACGACCACTTC 3' | 5' GTGAGTTTCCCGTTCAGCTC 3' | 202 |
| **miR-27a*** | 5' TTCACAGTGGCTAAGTTCCGC 3' | Universal q-PCR Primer | about 70 |
| **U6** | 5' CTCGCTTCGGCAGCACA 3' | 5' AACGCTTCACGAATTTGCGT 3' | 94 |

*Sense primers for mature miR-27a were provided here, anti-sense primer was provided by Invitrogen as Universal q-PCR Primer.
